# A Review of the Safety and Efficacy of Vaccines as Prophylaxis for *Clostridium difficile* Infections

**DOI:** 10.3390/vaccines5030025

**Published:** 2017-09-02

**Authors:** Mackenzie Henderson, Amanda Bragg, Germin Fahim, Monica Shah, Evelyn R. Hermes-DeSantis

**Affiliations:** 1 Ernest Mario School of Pharmacy, Rutgers, The State University of New Jersey, Piscataway, NJ 08854, USA; mh814@rutgers.edu (M.H.);amb602@rutgers.edu (A.B.); ehermesd@pharmacy.rutgers.edu (E.R.H.-D.); 2 Pharmacy Department, Monmouth Medical Center, Long Branch, NJ 07740, USA; monica.shah@rwjbh.org; 3 Pharmacy Department, Robert Wood Johnson University Hospital, New Brunswick, NJ 08901, USA

**Keywords:** *Clostridium difficile*, *Clostridium* infection, vaccination, toxoid vaccine, prophylaxis, disease prevention

## Abstract

This review aims to evaluate the literature on the safety and efficacy of novel toxoid vaccines for the prophylaxis of *Clostridium difficile* infections (CDI) in healthy adults. Literature searches for clinical trials were performed through MEDLINE, ClinicalTrials.gov, and Web of Science using the keywords bacterial vaccines, *Clostridium difficile*, and vaccine. English-language clinical trials evaluating the efficacy and/or safety of *Clostridium difficile* toxoid vaccines that were completed and had results posted on ClinicalTrials.gov or in a published journal article were included. Six clinical trials were included. The vaccines were associated with mild self-reported adverse reactions, most commonly injection site reactions and flu-like symptoms, and minimal serious adverse events. Five clinical trials found marked increases in antibody production in vaccinated participants following each dose of the vaccine. Clinical trials evaluating *C. difficile* toxoid vaccines have shown them to be well tolerated and relatively safe. Surrogate markers of efficacy (seroconversion and geometric mean antibody levels) have shown significant immune responses to a vaccination series in healthy adults, indicating that they have the potential to be used as prophylaxis for CDI. However, more research is needed to determine the clinical benefits of the vaccines.

## 1. Introduction

*Clostridium difficile* (*C. difficile*) is a gram-positive, spore-forming bacterium known for causing severe diarrhea [1]. *C. difficile* infections (CDI) are often contracted and transmitted in healthcare settings, such as hospitals and long-term care facilities [1,2]. Patients with prolonged hospitalizations are among those at the highest risk for developing CDI [1]. For these reasons, they are traditionally considered healthcare-related infections [3]. However, while most cases of CDI are contracted in healthcare settings, community-acquired CDI rates are increasing [1]. Other risk factors include antibiotic use, advanced age, cancer chemotherapy, and proton pump inhibitor use [1,4]. Antibiotic use increases the risk of CDI because of alterations in the normal bowel flora, providing a suitable environment for *C. difficile* to flourish [1]. Colonization of *C. difficile* results in an infection that can vary in degree of severity, ranging from mild inflammatory diarrhea to pseudomembranous colitis, toxic megacolon, sepsis, and death [2].

*C. difficile* exerts its effects on the gastrointestinal (GI) tract by releasing two toxins that can bind to and damage intestinal epithelium. Toxins A (an enterotoxin) and B (a cytotoxin) contribute differently to the pathophysiology of CDI. Toxin A is associated with the secretion of fluid and generalized inflammation in the GI tract. Toxin B is considered the main determinant of virulence in recurrent CDI, and is associated with more severe damage to the colon [2,3].

Since these two toxins are implicated in the harmful effects associated with CDI, they have become prominent targets in the search for preventative measures for the infection [5]. One major area of research involves the use of altered toxin structures as targets for a vaccine, termed toxoid vaccines. The toxoid is altered such that it will not damage the GI tract, but will prime the immune system to recognize and remove the real toxins from the bacteria if they are encountered in the future, thereby preventing disease [6,7,8]. There are a few toxoid vaccines for CDI currently in early stages of development, but there is not much evidence relating to their efficacy. Past research has shown a negative correlation between patients’ antitoxin antibody levels and their risk of recurrent CDI. For this reason, many researchers believe that a toxoid vaccine that can promote antibody production is a promising research pursuit [9].

While *C. difficile* toxoid vaccines can neutralize exotoxins and help prevent toxin-mediated symptoms, they also have several limitations. Previous research has indicated that toxoid vaccines do not have the ability to prevent colonization of C. *difficile* in the GI tract or prevent cytotoxicity [10]. Furthermore, they cannot prevent *C. difficile* sporulation or shedding of these spores into the environment, thus potentially increasing the number of asymptomatic carriers of the infection [11,12].

Several other types of *C. difficile* vaccines are also in development. For example, the approach of passive immunization from administration of monoclonal antibodies to both toxins A and B seems promising because of the long in vivo half-life of monoclonal antibodies [13,14]. A carbohydrate-based polysaccharide-II (PS-II)-conjugated vaccine directed toward *C. difficile* cell wall components has also demonstrated immunogenicity in animal models [12]. Vaccine candidates that inhibit colonization and adhesion of bacteria to gut epithelia are also being evaluated. In *C. difficile*, two proteins encoded by a single gene, *SIpA*, are found on the bacterial cell surface. An active vaccination regimen using an extract of two SIP proteins with different adjuvants has been shown to elicit an antibody response in animal models [12].

At present, methods for primary and secondary prevention of CDI are controversial. Many forms of prophylaxis have been proposed, including probiotics and antibiotics, though none are recommended by the Infectious Disease Society of America (IDSA). The only preventative measures currently included in the IDSA guidelines are antimicrobial stewardship and the maintenance of clean, disinfected surfaces to promote sanitary conditions [15].

The potential severity of and damage caused by CDI in combination with their rising incidence renders the subject of prophylaxis a pressing public health concern. Previous research into various toxoid vaccines suggests their promise as preventative measures and this review aims to evaluate clinical trials that test both the safety and efficacy of toxoid vaccines for CDI prophylaxis.

## 2. Materials and Methods

A literature search was devised to find research evaluating toxoid vaccines for the prevention of CDI. Outcomes of interest were safety measures (adverse reactions (ARs) and adverse events (AEs) experienced after vaccine administration) and efficacy measures. The trials found in this literature search assessed efficacy using several surrogate markers, including seroconversion as well as geometric mean fold rises (GMFRs) and concentrations (GMCs) of antibody levels. All methods for assessing efficacy were included.

A MEDLINE search (2000–2017) was performed using the key words bacterial vaccines and *Clostridium difficile.* Only clinical trials were included; trials not specifically performing research on the safety and/or efficacy of a *C. difficile* vaccine were excluded.

A ClinicalTrials.gov search (2000–2017) was performed using the key words “*Clostridium difficile* vaccine”. The search was limited to clinical trials that were closed and completed at the time of the search and had results posted. One trial that did not have results posted was included because the results were published in a corresponding journal article.

A third literature search was performed for articles through Web of Science (2000–2017) using the keywords *Clostridium difficile* toxoid vaccine. Only clinical trials were included.

Six clinical trials were included in this review. Out of 85 primary results from the MEDLINE search, two trials met the inclusion criteria once duplicates were excluded. From 17 found in the initial ClinicialTrials.gov search, 12 were completed at the time of the search. Four of these met all the inclusion criteria. One clinical trial included in the review had results posted on ClinicalTrials.gov that have not been published [16]. Out of the eight primary results found through Web of Science, none met the inclusion criteria for this review.

## 3. Results

Every trial evaluated a *C. difficile* vaccination series (3–4 doses of vaccine) administered to healthy participants at pre-specified time points over varying intervals (ranging from 21–180 days). Five trials [17,18,19,20,21] used two variations of a toxoid vaccine: an aluminum-based adjuvant (Alum) vaccine and a non-Alum vaccine, the sixth trial [16] did not specify the formulation. In every trial, the researchers followed up with participants at one or more time points to collect blood samples and self-reported safety data. All trials utilized a dose-escalating treatment regimen, in which participants were assigned to receive different doses of the vaccine.

To assess safety, the trials looked at local and systemic ARs and AEs experienced after vaccination. Every trial measured safety endpoints for at least six days after each dose, the most common period of measurement being seven days after each dose. All trials reported mild local ARs. Overall, there were few moderate/severe ARs or AEs reported. The most commonly reported ARs/AEs were related to the injection site (e.g., pain, erythema) and flu-like symptoms (e.g., malaise, fatigue, headache). Detailed safety information from each trial is outlined in Table 1. 

In addition to safety endpoints, five trials evaluated efficacy endpoints [17,18,19,20,21]. The main efficacy parameter was immune response to vaccination in the form of anti-toxin antibody production. The specific methodology used to measure efficacy outcomes in each trial is summarized in Table 2. Seroconversion was defined as an increase in antibody levels of at least four times the baseline value (≥4-fold increase from baseline). GMFR uses the average fold-increase in log transformed antibody levels from baseline. GMCs use the absolute average of the log transformed antibody levels. Seroconversion rates and GMFRs are summarized in Table 2.

### Summary of Trials

Kotloff et al. [17] assessed the safety, immunogenicity, and dose response of a *C. difficile* toxoid vaccine as Alum or non-Alum formulations, in 30 healthy participants (median age: 23 years). Individuals were excluded from the trial if they had a history of antibiotic-associated diarrhea or antibiotic use in the past month. Participants were sequentially assigned to receive one of three study doses: 6.25, 25, or 100 mcg. Vaccines were administered on day 1, 8, 30, and 60. The researchers assessed IgA and IgG antibody production by collecting peripheral blood mononuclear cells (PBMC) from participants one week before and after immunization and performing an adapted Enzyme-Linked Immunospot (ELISPOT) assay on the collection. In the 6.25 mcg dose group, two participants (20% of the group) did not exhibit ≥4-fold rises in antibodies. One of the participants that did not respond received an Alum adjuvant vaccine and the other received a non-Alum vaccine. The highest antibody responses were seen in the 25 mcg with Alum and 100 mcg non-Alum dose groups for Toxin A. In contrast, antibody responses increased with increasing dose for Toxin B. Vaccine dose and formulation were not found to be significantly related to the magnitude of antibody response. Levels of serum antibodies were found to correlate with serum anti-Toxin A IgG (*r* = 0.83, *p* < 0.001). The researchers reported that a three dose series (on day 1, 8, and 30) appeared to be adequate because a fourth dose did not substantially boost serum IgG or antibodies.

Bezay et al. [18] evaluated VLA84 (experimental vaccine), a single recombinant fusion protein comprised of portions of *C. difficile* Toxins A and B. The primary outcomes were safety and tolerability of VLA84 in healthy adults using different doses and formulations. The secondary outcomes were immunogenicity and dose-response. In part A of the study, 60 healthy adults (18–64 years) received one of five vaccine doses: VLA84 20 mcg Alum, 75 mcg Alum or non-Alum, or 200 mcg Alum or non-Alum. Three doses of the vaccine were given on day 0, 7, and 21. In part B of the study, 80 elderly (≥65 years) participants were randomized to receive VLA82 75 mcg Alum or non-Alum or 200 mcg Alum or non-Alum formulations on day 0, 7, 28, and 56. The researchers assessed antibody production by collecting serum samples from participants on day 0 and 28 (adults) or 56 (elderly) and performing Enzyme-Linked Immunosorbent assays (ELISA) and Toxin Neutralization Assays (TNA) on the collections. The researchers reported high antibody responses to both toxins in both the adult and elderly groups after vaccination. However, the 20 mcg Alum dose group showed significantly lower responses than the 75 and 200 mcg non-Alum dose groups at several time points. The highest antibody responses were found one week after the last vaccination in the adult sample and four weeks after the last vaccination in the elderly sample. Based on preliminary analysis, the researchers chose to analyze antibody responses for the 75 mcg non-Alum dose group. The results of this analysis are summarized in Table 2. In this dose group, antibody responses to both *C. difficile* toxins were found to decrease to 20–25% of the peak response after six months, similar to past findings [19].

Greenberg et al. [19] assessed an adjuvant *C. difficile* toxoid vaccine in adult (18–55 years) or elderly (≥65 years) participants over a 70-day treatment period. Participants with a history of antibiotic-associated diarrhea or recent antibiotic use were excluded. Participants were randomly assigned to one of four groups: 2, 10, or 50 mcg or placebo. Three doses of the vaccine were administered on day 0, 28, and 56. The researchers assessed IgG production to both toxins A and B by collecting serum samples from participants and obtaining ELISA titers on the collections, using previously collected pooled human plasma as a control measure. The fastest antibody level increase rates were reported for the 50 mcg dose group. In both adult and elderly participants, antibody responses for Toxin B were found to be lower than for Toxin A. Anti-Toxin A and B antibody levels were found to increase until day 70 and were in decline by day 236, which is consistent with responses to other types of toxoid vaccines [22].

de Bruyn et al. [20] assessed the optimal formulation and dosing schedule for an investigational *C. difficile* toxoid vaccine. To be included in the trial, participants had to be considered at increased risk for infection, which was defined as impending hospitalization and/or prolonged residence in a long-term care facility or nursing home. In stage 1 of the study, 455 participants were randomized to one of five groups: 50 or 100 mcg Alum or non-Alum formulations or placebo. Participants received three doses of the vaccine on day 0, 7, and 30. In stage 2 of the study, 206 participants were randomized to receive a selected vaccine formulation, based on immunogenicity seen in stage 1, according to two vaccination schedules (day 0, 7, and 30 or day 0, 30, and 180). The researchers assessed anti-toxin IgG to toxins A and B as well as anti-toxin neutralizing capacity by collecting blood samples from participants and obtaining, respectively, ELISA titers and TNA titers on the collections. GMCs for both toxins were found to peak on day 60 and decline by day 180. A comparison of the immune responses based on the formulation and schedule resulted in the 100 mcg plus Alum formulation and day 0, 7, and 30 schedule being selected for further development. From the trial results, the researchers reasoned that a vaccine formulation that included an adjuvant would be critical to enhance immune responses in high-risk patients.

Sheldon et al. [21] evaluated a three-dose series of a *C. difficile* toxoid vaccine. The primary outcome was the safety and tolerability of the *C. difficile* vaccine. The secondary outcome was immunogenicity of the vaccine, measured by GMFRs of *C. difficile* Toxin A-specific and Toxin B-specific antibodies. Anyone with a history of CDI or antibiotic use in the past month was excluded from the trial. Participants were randomly assigned to one of seven groups: 50, 100, or 200 mcg Alum or non-Alum formulations or placebo. Vaccines were administered on day 1, 30, and 168. Two cohorts of participants were studied. Cohort 1 consisted of adults 50–64 years of age (*n* = 97) and cohort 2 consisted of adults 65–85 years of age (*n* = 95). The researchers assessed neutralizing antibody production by collecting serum samples from participants and performing TNAs on the collections. Substantial increases in antibodies from baseline to both toxins A and B were found in vaccinated participants one month after the second dose and one month after the third dose. The researchers reported that the increases in antibody levels after seven months persisted at least 12 months. Antibody levels for both toxins tended to be higher for the non-Alum compared to the Alum formulations at nearly all time points. No dose–response relationship was observed for vaccine doses and formulations, which the researchers noted may be due to the trial’s lack of power. 

Finally, a national clinical trial evaluated local and systemic vaccine-related ARs to a *C. difficile* toxoid vaccine, but did not evaluate efficacy endpoints [16]. As previously mentioned, this clinical trial has safety results posted on ClinicalTrials.gov, but these results have not been published. 

## 4. Discussion

As research into toxoid vaccines for the prevention of CDI builds, it is essential to compile the findings of individual trials to understand the progression of the field of research as a whole. This review assessed the methodology and outcomes of six clinical trials that tested the safety and efficacy of toxoid vaccines to prevent CDI.

Every trial included in this review found mild ARs consistent with those found to accompany vaccine administration in general [23]. From these findings, toxoid vaccines seem promising from a tolerability and safety standpoint. However, two trials specified that participants were only able to report ARs that were pre-specified by the researchers [16,17]. These ARs tended to be those well-documented to be associated with vaccines (such as injection site pain, fever, and headache). This methodology may have biased participant reporting and resulted in researchers overlooking less common ARs or AEs from the vaccine. Future research should maintain focus on the safety of the vaccine, particularly as more participants and patients become involved in later phases of research.

An interesting finding from multiple studies was the absence of a dose-response relationship to the vaccine for both safety and efficacy. There was no clearly demonstrated or consistent relationship between increasing doses of the vaccine and ARs or AEs, or between increasing doses of the vaccine and the body’s immune response. It is not practical to directly compare the trials’ vaccine regimens to determine optimal dosing because the formulations and vaccines themselves differed. However, the best dosing of the vaccines included in this review are as yet unclear and more research is required to determine the lowest doses that provide the optimal immune response and minimized side effects.

Three trials found that antibody responses were notably diminished by 145–160 days after the last vaccine [18,19,20]. de Bruyn et al. [20] tested different dosing schedules and their findings suggest that the timing of vaccine administration may play an important role in the body’s response to vaccination. The results found in the trials in this review question the vaccines’ long-term effects, and bring up the potential need for further doses or boosters of the vaccine to allow patients to experience long-term clinical benefits. 

The trials that evaluated the use of Alum adjuvants with their vaccines did not find clear relationships between the use of an adjuvant and any outcome measures; while two trials [18,21] found the non-Alum formulations resulted in higher immune responses, one trial [20] found that the Alum formulations resulted in higher immune responses. Adjuvants are generally used with vaccines to increase the body’s immune response, but are often associated with a greater degree of ARs and AEs [24]. The trials in this review did not reliably find an increasing immune response or rate/severity of adverse reactions for adjuvant vaccines compared to non-adjuvant vaccines.

Five trials differentiated between participants’ immune response to Toxin A versus Toxin B [17,18,19,20,21]. All of these trials found increases in antibodies to both toxins at various time points after vaccine administration. However, the findings did not demonstrate whether there were significant differences in magnitude between antibodies produced to Toxin A or Toxin B, and if so, which had a stronger response. As previously mentioned, while both toxins contribute to the pathophysiology associated with CDI, Toxin B is generally associated with worse outcomes. Therefore, an optimal *C. difficile* vaccine may need to account for this, targeting antibody production against Toxin B over Toxin A if possible. From the results of the trials included in this review, there was no reliably directed response toward one toxin over the other. These are important points for further research, especially considering the different ways the toxins contribute to the clinical manifestations of the disease. 

Finally, primary and recurrent/secondary CDI are both serious public health concerns and need to be considered to fully understand the scope of the infection. Though vaccines for recurrent CDI have not been evaluated in clinical trials, Sougioultzis et al. [25] found that six months after the last dose of a four-dose vaccine series, three patients with recurrent infection showed no further recurrence. However, antibody responses to vaccination in these three patients were variable; only two showed substantial increases compared to baseline. These results highlight the point that surrogate endpoints used as efficacy measurements are not sufficient to claim efficacy or effectiveness of the toxoid vaccines. 

Bezlotoxumab is a monoclonal antibody that has been studied more extensively in patients who have experienced CDI. When given in conjunction with antibiotic treatment for a *primary* CDI, bezlotoxumab significantly reduced the risk of developing recurrent/secondary CDI [26]. Since this drug has been shown to be clinically effective in the target population, it calls into question what the appropriate timing to administer toxoid vaccines would be and their place in therapy.

## 5. Conclusions

Though a substantial amount of research has been done on *C. difficile* toxoid vaccines in recent years, there are still many areas of inconsistency in the literature. While the vaccines have been shown to be generally well tolerated, their efficacy is questionable. All trials in this review that evaluated efficacy found substantial immune responses after vaccination. However, there were no clear relationships between dose and response or between adjuvant formulation and response. Furthermore, there is evidence that antibody levels from the vaccine may diminish in the long term. These points of contention show the need for further research on the optimal dose, dosing schedule, and formulation of the toxoid vaccines. Finally, the efficacy of the vaccines seems promising from surrogate endpoints measured in these clinical trials, but more research is needed to determine their clinical benefits.

## Figures and Tables

**Table 1:** Safety outcomes most commonly reported by participants.

Trial	Collection Period (Days)	ARs and AEs Reported
Kotloff et al. [17]	7 (after each dose)	Rash (26.7%) Pain at injection site (60%; more common with adjuvant, *p* = 0.004) Abdominal pain (20%) Malaise (16.7%) Swelling & erythema (increased with dose; *p* < 0.001, *p* = 0.04)
Bezay et al. [18]	7 (after each dose)	18–65 cohort – Pain at injection site (21%, *p* = 0.001) ^1^
Greenberg et al. [19]	7 (after each dose)	18–55 cohort – Pain (85–100%), erythema (42–50%), swelling (15–25%) & induration (8–33%) at injection site ≥65 cohort – Pain (33–67%) & erythema (8–25%) at injection site Increased eosinophil count (25–42%) Fatigue (17–25%)
de Bruyn et al. [20]	ARs: 6 (after each dose) AEs: 30 (after each dose)	Pain at injection site (42.4–68.3%) Myalgia (33.3–45%) Malaise (29–33.7%) Headache (27.3–35.6%) Arthralgia (20.2–30%)
Sheldon et al. [21]	ARs: 7 (after each dose) AEs: 365 (after 1st dose)	50–64 & 65–85 cohort Pain at injection site (lasting 1–2 days) Headache (1–3 days) Fatigue (1–3 days) Upper respiratory tract infection
Pfizer [16]	7 (after 1st dose) 14 (after 2nd, 3rd dose)	50–64 cohort ^2^ Pain (16.7–66.7%), erythema (5.6–50%) & swelling (16.7–33.3%) at injection site Headache (5.6–33.3%) Fatigue (11.1–38.9%) 65–85 cohort ^2^ Pain (24.6–66%), erythema (6.7–30%) & swelling (3.3–26.4%) at injection site Headache (8.3–26.4%) Fatigue (8.3–39.6%) New/worsening muscle or joint pain (0–26.4%)

^1^ This was the only AR/AE reported with a *p*-value of ≤ 0.05; ^2^ ARs/AEs reported >10% more frequently by participants in vaccine groups than placebo groups.

**Table 2:** Summary of major efficacy outcomes.

Trial	Trial Design	N	Participant Age (Years)	Efficacy Results
Kotloff et al. [17]	Sequential assignment, double-blind, phase 1 trial	30	23	**Seroconversion Rates:** 100% of participants in the 25 and 100 mcg dose groups 80% of participants in the 6.25 mcg dose group
Bezay et al. [18]	Multi-center, open label, partially randomized, phase 1 trial	140	30.8 (Part A) 68.3 (Part B)	**Seroconversion Rates** (for 75 mcg non-Alum dose group): **Part A**––By day 28: 70% of participants and 80% of participants (to Toxin A and B, respectively) **Part B**––By day 56: 91% of participants and 55% of participants (to Toxin A and B, respectively)
Greenberg et al. [19]	Two randomized, placebo-controlled, double-blind, phase 1 trials	98	18–55 (cohort 1) ≥65(cohort 2)	**Seroconversion Rates:** **Cohort 1**––By day 56: 100% of participants who received any dose of vaccine **Cohort 2**––By day 56: 50%, 89%, and 100% of participants who received any dose of vaccine (2, 10, 50 mcg, respectively) **Placebo**––0% of participants at all time points
de Bruyn et al. [20]	Multi-center, Placebo-controlled, randomized, phase 2 trial performed in 2 stages	661	40–64 (cohort 1) 65–75 (cohort 2)	**Highest Seroconversion Rates:** By day 60: 97% of participants who received 100 mcg with Alum By day 60: 93.2% of participants who received 100 mcg non-Alum
Sheldon et al. [21]	Placebo-controlled, randomized, observer-blinded phase 1 trial	192	50–64 (cohort 1) 65–85 (cohort 2)	GMFRs: **Cohort 1**––By 7 months (day 210): GMFR = 59–149.23 and 116.67–2503.75 compared to 2.47 and 2.48 in placebo groups (to Toxin A and B, respectively) **Cohort 2**––By 7 months (day 210): GMFR = 42.73–254.77 and 136.12–4922.8 compared to 2.03 and 1.58 in placebo groups (to Toxin A and B, respectively)
